# Development of interactive dashboards for monitoring endemic animal pathogens in Ontario, Canada: Ontario interactive animal pathogen dashboards

**DOI:** 10.1177/10406387231190076

**Published:** 2023-08-04

**Authors:** Tatiana Petukhova, Maria Spinato, Tanya Rossi, Michele T. Guerin, David Kelton, Pauline Nelson-Smikle, Melanie Barham, Davor Ojkic, Zvonimir Poljak

**Affiliations:** Department of Population Medicine, Ontario Veterinary College, University of Guelph, Guelph, Ontario, Canada; Animal Health Laboratory, University of Guelph, Guelph, Ontario, Canada; Animal Health Laboratory, University of Guelph, Guelph, Ontario, Canada; Department of Population Medicine, Ontario Veterinary College, University of Guelph, Guelph, Ontario, Canada; Department of Population Medicine, Ontario Veterinary College, University of Guelph, Guelph, Ontario, Canada; Animal Health Laboratory, University of Guelph, Guelph, Ontario, Canada; Animal Health Laboratory, University of Guelph, Guelph, Ontario, Canada; Animal Health Laboratory, University of Guelph, Guelph, Ontario, Canada; Department of Population Medicine, Ontario Veterinary College, University of Guelph, Guelph, Ontario, Canada

**Keywords:** cattle, data, dashboard, livestock, poultry, swine, turkeys, visualization

## Abstract

The advancement of web-based technologies makes it possible to build user interfaces or web pages that present and summarize complex data in easy-to-read graphical formats that emphasize key information. Taking advantage of this technologic progress, we addressed the need for real-time visualizations of trends for major pathogens in the largest livestock industries in Ontario: poultry, swine, and cattle. These visualizations were built using test data from the laboratory information management system of the Animal Health Laboratory at the University of Guelph, a large veterinary diagnostic laboratory in Ontario. The data were processed using R software and used to construct interactive and dynamic visualizations using Tableau Desktop v.2021.4 (Tableau Software). We designed 12 dashboards: in chickens—influenza A virus, fowl adenovirus, infectious bronchitis virus, and infectious laryngotracheitis virus; in turkeys—influenza A virus; in swine, influenza A virus, rotavirus, and porcine reproductive and respiratory syndrome virus; in cattle—bovine viral diarrhea virus, *Mycoplasma bovis*, *Salmonella* Dublin in individual samples, and *Salmonella* Dublin in bulk tank milk samples. Data for each pathogen are presented in 2 dashboards. One shows the data of the last 10 y (general view) and the other the data of the last 3 y, but in more detail (comprehensive view). Information on gaining access to all dashboards is available at https://iapd.lsd.uoguelph.ca/. The visualizations provide near-real-time access to aggregated assay results for selected pathogens for veterinarians, animal health regulatory agencies, researchers, and other users who are interested in livestock pathogen surveillance.

Definitions of disease monitoring in animal and human populations differ among sources,^6,14,16,25,26,28,30^ but commonly include statements about ongoing efforts to assess the disease status of a given population.^30^ Traditionally, for animal diseases, surveillance has referred to systems in which reaching a predefined level of disease prevalence can trigger some directed actions to improve population health.^30^ In public health, definitions have evolved and have been reviewed elsewhere.^2,3,25,27,28^ The 1986 definition of surveillance by the U.S. Centers for Disease Control and Prevention (CDC) states that it is “an ongoing systematic collection, analysis, and interpretation of health data,” and is “closely integrated with the timely dissemination of these data to those who need to know.”^30^

The advancement of web-based technologies allows comprehensive utilization of surveillance. The improvement and accessibility of these technologies have contributed to widespread implementation of tools that make it possible to visualize not only disease trends over time but also spatial distribution and other surveillance metrics. The need for real-time visualization of disease trends has been heightened during the COVID-19 pandemic and the 2022 monkeypox outbreak.^10,12,23,29^ The designed reporting systems relied on the delivery of accurate and accessible public health data within an expected time frame. As new data were collected, the outcomes were automatically analyzed and integrated, offering near-real-time insights. Consequently, modeling and analysis of these data provide informative data insights, improve surveillance, facilitate informed decision-making, and enable evidence-based public health policy to reduce risks and save lives.^10,12,23,29,30^

In animal health, several interactive visualization platforms have been developed and reported in the scientific literature, including the Iowa State University surveillance system.^11^ Ontario is a leading Canadian province in agriculture and livestock production.^4,17,25^ Veterinarians regularly use diagnostic laboratories for diagnostic investigation, disease monitoring, certification, and export purposes. Most of the testing for livestock diseases and pathogens in Ontario is conducted at the Animal Health Laboratory (AHL; University of Guelph, Guelph, Ontario, Canada), one of the largest animal health diagnostic laboratories in Ontario, which maintains a laboratory information management system (LIMS), a database that contains test results for animal pathogens. Detailed results of tests are provided to the clients who submit samples. An aggregated summary of testing is communicated to a variety of end users, representing animal and public health organizations and industry groups, through a variety of channels, including discussions during Ontario Animal Health Network (OAHN) meetings, newsletters, industry reports, and presentations. Given the availability of advanced technologies, the state of the database, and the end users’ expectations, it would be beneficial to develop dashboards that would allow interactive visualization of disease trends for major livestock pathogens in real- or near-real-time.

Our overall goal was to develop a dynamic and interactive tool that would allow near-real-time processing, integration, visualization, and analysis of data for pathogens in 3 livestock populations (poultry, swine, cattle) that account for the highest animal economic output in Ontario. For instance, data from Statistics Canada indicate that, in 2021, the total number of hen and chicken farms in Ontario was 8,051^24^; those farms raised over 53 million animals per year.^24^ Ontario is also home to 683 turkey farms that raise over 2.4 million turkeys per year.^24^ Ontario chicken farmers contribute over $4.2 billion in economic activity each year.^5^ The Ontario pork industry represents 2,437 farms,^24^ marketing 7.1 million pigs annually in the province,^1^ which generates ~$3 billion of Ontario economic output.^18^ The dairy cattle industry in Ontario has 3,273 dairy farms, producing over 3 billion L of milk annually; the industry contributed more than $7.7 billion to the provincial gross domestic product in 2022.^7^

The pathogen data consisted of PCR and ELISA results of diagnostic and monitoring procedures that have been available historically at the AHL and are being generated continuously. Herein, we describe our development of dashboards for pathogens in poultry, swine, and cattle.

## Materials and methods

### Needs assessment: eliciting opinions of veterinary practitioners and other animal health professionals

For the initial phase of our project, the focus and the target audience were animal health experts working in the 3 major livestock industries of Ontario. To better understand the needs of the target populations, the research team organized focus groups with health experts from the 3 respective industry groups. Focus group participants were recruited through 2 methods: the OAHN co-leads, and research team invitations. More information about OAHN can be found elsewhere^15^ and on their website (www.oahn.ca). Briefly, each animal species group has an expert network of veterinarians from private practice, the Ontario Veterinary College, the AHL, and the Ontario Ministry of Agriculture, Food and Rural Affairs, with additional veterinarians and industry members for some species networks. Members of the networks meet every quarter to discuss and interpret laboratory data, review clinical observations from veterinary surveys and members directly, and ultimately, discuss implications for animal health. In September 2020, OAHN co-leads of the 3 relevant livestock industries invited members of the OAHN expert networks to participate in focus groups to inform the development of dashboards, in addition to external experts. The additional invitations were extended based on the areas of expertise within academia, government, and private stakeholder groups to provide a wide breadth of experience.

A total of 15 experts participated in the focus group meetings.^13^ There were 3 meetings in early October 2020. Each meeting was held virtually using the Zoom platform for 1.5 h and was recorded. The focus groups used qualitative data-collection techniques. Similarities and needs mentioned at least twice were included in a report to streamline and understand common interests and then were used to inform project directions and select pathogens of interest. Discussed ideas were organized by the following themes: 1) user experience and visual appearance; 2) use of data and outputs; 3) end users, access to data, and integration with current systems; 4) pathogens of immediate interest; and 5) assessment of the need for disease forecasting.

The focus group questions and methodology were reviewed and approved by the University of Guelph Research Ethics Board (REB approval 20-06-032). Results were transcribed using Otter (Otter.ai).

### Diseases and pathogens

**Figure 1.**
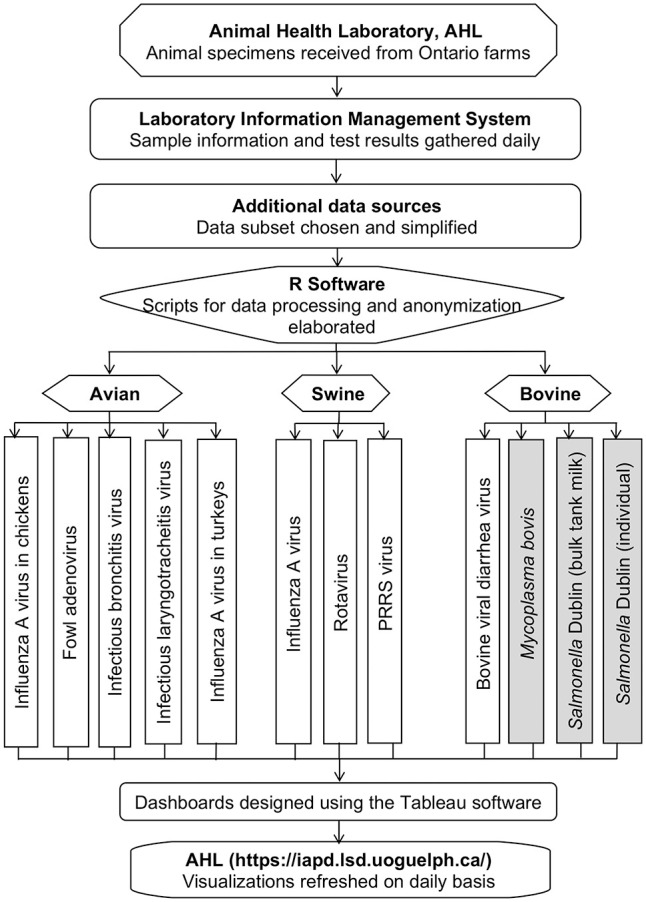
Sequential steps of data processing for detection, monitoring, and visualization of important livestock pathogens. Shaded boxes (*Mycoplasma bovis*, *S*. Dublin) indicate bacterial pathogens. PRRS = porcine reproductive and respiratory syndrome. Poultry, swine, and cattle samples were received from Ontario farms and were tested in the Animal Health Laboratory (AHL; University of Guelph, Ontario, Canada). Sample information and test results were stored in the laboratory information management system (LIMS). Pathogen data were retrieved and processed using R software. The final optimized, anonymized, and aggregated datasets for each pathogen were used to build visualizations in Tableau software. The constructed dashboards were uploaded to the Ontario interactive animal pathogen dashboards website and were embedded in the visualization platform. Newly generated test result data are integrated daily from the LIMS into R-scripts, and the dashboard visualizations are refreshed on scheduled daily runs.

For the development of initial dashboards, the following combinations of pathogens and hosts were selected based on focus group input (Fig. 1): in chickens—influenza A virus (IAV; *Alphainfluenzavirus influenzae*), fowl adenovirus (FAdV; *Fowl aviadenovirus A–E*), infectious bronchitis virus (IBV; *Avian coronavirus*), infectious laryngotracheitis virus (ILTV); in turkeys—IAV; in swine, IAV, rotavirus, porcine reproductive and respiratory syndrome virus (PRRSV; *Betaarterivirus suid 1*, *Betaarterivirus suid 2*); in cattle—bovine viral diarrhea virus (BVDV; *Pestivirus bovis*, *Pestivirus tauri*), *Mycoplasma bovis*, and *Salmonella enterica* subsp. *enterica* ser. Dublin (*S.* Dublin) in individual samples of cattle, as well as *S.* Dublin in bulk-tank milk samples.

### Data source

The AHL receives various types of animal samples for diagnostic, monitoring, certification, research, and other purposes, sourced from animal populations in Ontario. Animal samples are usually accompanied by basic demographic and clinical data. Individual samples for pathogens included in our project were evaluated using various PCR assays. The only pathogen that was evaluated using ELISA was *S.* Dublin in cattle. The obtained sample information and test result reports were recorded as submission- and specimen-level data. The submission-level data contained reasons for submission, species, and demographic and clinical data; specimen-level data included results from PCR assays and ELISA. All data were stored in the AHL LIMS and used for developing dashboards (Fig. 1).

### Data structure

Pathogen data stored in the AHL LIMS were large and complex, containing the results of assays, and could be reported as qualitative or quantitative results. To ensure inclusion of all relevant test results, standardization of assays to be included was achieved through assigning tags (e.g., variable DISEASE), and the same assay result could have one or more tags, depending on how the assay was used. For example, any type of PCR assay that was used to screen for IAV would be tagged as both “influenza A PCR” and “influenza A virus,” whereas PCR assays that were used for subtyping would be tagged as “influenza A PCR typing” and “influenza A virus.” This is because all of the tests represented some form of assessment for IAV, but the typing PCR for IAV used in the laboratory was sequential in nature, following the positive IAV PCR assay in most situations. For some pathogens (e.g., porcine rotavirus), the tagging system was assigned differently because multiplex PCR assays that were used for rotavirus speciation replaced a general PCR test for rotaviruses. Therefore, assays were used in parallel, and any finding of a positive result on a species-specific PCR would also indicate a rotavirus-positive finding. Consequently, such pathogen species–level PCR assays were tagged as “rotavirus,” “rotavirus PCR,” and “rotavirus PCR typing.”

Among variables available from the pathogen data, we selected those that included temporal, demographic, clinical, laboratory, signalment, geospatial, and species information (Table 1).

**Table 1.** Description of the selected pathogen data fields. Records from the tested animal samples in the Animal Health Laboratory (AHL; University of Guelph, Ontario, Canada) were stored in the AHL laboratory information management system. The variables included temporal, demographic, clinical, laboratory, signalment, geospatial, and species information.

Field	Description	Details
SUBMISSIONDATE	The date when a submission was created in the laboratory information management system	Standardized for all pathogens (e.g., year-month-day)
SUBMISSIONID	A unique set of characters assigned to each submission to identify included samples during transportation to laboratory sections, testing, and reporting	yy-xxxxxx
SAMPLEID	A unique set of characters assigned to each sample and related to a unique submission ID. A test result is linked with a sample ID	yy-xxxxxx-001
SAMPLETYPE	A type of sample	Different for each sample ID (e.g., swab, tissue, milk, blood)
SPECIES	An animal species	e.g., porcine, bovine, turkey, chicken
COMMODITY	Specific demographic or production category within a species	Different for each species (e.g., porcine: suckling, nursery/weaner, grower/finisher, gilt/sow, boars, other [not specified])
CASETYPE	A purpose for testing	Standardized for all pathogens (except *Mycoplasma bovis*) and linked with a specific submission form (e.g., diagnostic, monitoring, other)
GROUPOFTEST*	A description of a diagnostic test	Different for each species and each pathogen (e.g., influenza A virus, avian - rt-RT-PCR, influenza A, matrix PCR, swine - real-time PCR)
DISEASE†	A standardized description of a pathogen detected, assay used to detect the pathogen (or exposure to it), and species/subtype/genetic grouping used for tagging and data retrieval	e.g., influenza A virus, influenza A virus PCR
DISPLAYVALUE	Reported quantitative or qualitative results from testing	Standardized for all pathogens (e.g., negative, not detected, inconclusive, suspicious, weak-positive, insufficient, not analyzed)
PROVINCE	A description of a province of Canada	e.g., Ontario

* Other variables with the description of tests in the AHL laboratory information management system were not included in this table as those variables provided similar descriptive information regarding performed tests as the GROUPOFTEST variable.

† Each observation could have more than one value, depending on the nature of the assay and its use in a testing protocol.

### Data processing

Scripts were developed in R v.4.1.1^22^; data processing included pipelines that executed automated database querying and data import, filtering, cleaning, organization, standardization, anonymization, and aggregation of data. The R packages used to facilitate this process were: odbc, DBI, dplyr, tidyr, stringr, lubridate, scales, data.table, doBy.^22^

Each tested laboratory submission consisted of one or more samples. A submission was considered to have been processed if at least one test for PCR testing or ELISA (for *S*. Dublin) for a pathogen was requested. Processed submissions were included if they were tested for diagnostic, monitoring, and other purposes; submissions tested for research studies were excluded. Outcomes from the included submissions were refined and aggregated to obtain the number of unique submissions and the number of unique positive submissions. That is, observations with reported quantitative test results (e.g., expressed as Ct values) were excluded to avoid duplication. The format of qualitative display values from testing was changed from descriptive to dichotomous, in which outcomes reported as “negative,” “not detected,” “inconclusive,” “suspicious,” or “weak-positive” were considered negative, or 0; otherwise, positive, or 1. Test result outcomes interpreted as “insufficient” and “not analysed” were characterized as “not available” and treated as missing values in logical computation procedures. A submission was treated as positive if at least one sample within the submission was declared as positive.

The smallest temporal unit in the data-aggregation process was one week. Submission dates were converted into weekly intervals; a week was considered to run from Sunday to Saturday. Then, records were aggregated at the submission date (i.e., the date when the week started), submission ID, case type, and commodity level. To build dashboards, the subsets of the processed datasets were stored in the AHL LIMS, including only the last 10 y.

### Database connection and dashboards

The final optimized, anonymized, and aggregated datasets from the AHL LIMS were connected to the Tableau server and made available to the Tableau software using supported connectors, and the data fields were connected to the data source with a live connection to directly update any changed fields.

The variables from the datasets were integrated into worksheets for visualizations. A worksheet is a file in which variables are organized and used to create either a single chart or a table. Variables were also used to create new calculated fields and new columns in the data source. That is, the numbers of submissions and positive submissions were displayed with stacked vertical bar charts. The percentage of positive submissions over the total number of submissions was calculated and displayed in vertical bar graphs. Simple moving averages for the percentage of positive submissions were computed based on previously specified time periods to smooth short-term variation, eliminate possible seasonal effects, and highlight long-term trends and cycles. The percentage of positive submissions and estimated moving averages were displayed in dual charts with a scatter plot representing the percentage of positive submissions and a line representing the moving average values.

To make visualizations more informative, interactive, and dynamic, we created several customization parameters and added dashboard features. A date parameter was created to allow users to view results at their preferred level of time aggregation: weeks, months, quarters, or years. A rolling average parameter was also constructed to calculate moving averages based on the previous 2, 6, 8, 12, and 16 wk and, thereby, allow users to select the amount of smoothing. In addition, a dynamic Year-to-Date calculator was created to summarize the number of submissions, positive submissions, and the percentage of positive submissions. These parameters facilitated the production of interactive and dynamic visualizations that can be customized to suit users’ needs and make comparisons across case types, time periods, and commodities easy.

Dashboards were made up of multiple worksheets, each displaying different aspects of the data. For example, all dashboards include worksheets using the previous 10 y of lab data to show general data insights and long-term trends at a lower resolution and similar worksheets displaying data for the past 3 y with some features of dashboards modified to make visualizations more comprehensive. Then a sequence of dashboards was created in a 2-point story, where each story point represents a pathogen dashboard.

### Features of dashboards

Each pathogen dashboard has the following set of features: filters to display the aggregated results over time by date level, case type, and commodity type; highlights in labels to emphasize results by brightening specific marks and dimming all others; tooltips to reveal more details about the relevant data by hovering over a mark; interactivity with visualizations based on parameter values; and interactivity with visualizations by selecting marks or hovering.

### Visualization of dashboards

The final visualization versions were reviewed by collaborators to reach a consensus on how best to communicate the information and present charts clearly and efficiently in a story. Furthermore, a beta-testing disease dashboard and a survey were designed and sent to beta-testers for the dashboard assessment to determine if the designed format was easily understood and useful for veterinarians, industry, and regulatory stakeholders. Based on the comments from the collaborators and the beta-testers’ feedback, the dashboard features were adjusted to meet suggested recommendations and needs.

The final versions of constructed dashboards were uploaded to the Ontario Interactive Animal Pathogen Dashboards (IAPD) website, and the dynamic and interactive stories were embedded in the visualization platform. A 2-point story can be accessed under a pathogen dashboard at https://iapd.lsd.uoguelph.ca/. Access to the site is available for everyone who has Internet access. However, near-real-time dashboard visualizations are securely stored and can be accessed only with an approved login account.

## Results

### Focus group

Bovine, porcine, and poultry focus groups were formed based on their primary OAHN species network affiliation. Five topics discussed during the focus group meetings reflected the following actions.

For *user experience and visual appearance*, many participants expressed the need for a temporal graphic representation of the data, and spatial mapping was desirable. For the latter, there were different opinions regarding the level of granularity. Some participants expressed a preference for assessing individual premises, whereas others preferred information at the county level. The majority suggested geographical representation overlaid with publicly available information such as weather, as long as client confidentiality could be protected. Consistently, drill-down options were preferred, with livestock species, commodity, pathogen, and time period as options that were specifically listed. Some study participants expressed a preference to be able to display weather and other publicly available environmental data as an option. Browser-based solutions were considered potentially challenging when in rural areas, and purpose-designed apps were considered desirable by some practitioners. Practitioners also indicated that they would use the application sometimes on a laptop or desktop computer in their offices, and sometimes on a laptop on a farm with a client or before a consult on a farm to help inform decision-making. Study participants emphasized the need for dashboards to load quickly, as well as consideration of data usage by mobile users.

For *use of data and outputs*, veterinary practitioners in some sectors expressed the desire to download and manipulate the data within their own production systems. Similarly, stratifying data by individual clinic was expressed as important.

For *end users and for access to data and integration with current systems*, some practitioners independently voiced that they would like to have the dashboard interface with current options available to AHL clients (e.g., client portal) without a separate login. Although opinions varied, most felt that veterinarians would be the end users of these data at this time.

For *pathogens of immediate interest*, study participants identified different pathogens as priority. The following pathogens were noted by >2 participants in each sector. In the swine sector, PRRSV, porcine epidemic diarrhea virus (PEDV), and IAV were identified as priority pathogens. In the poultry sector, reovirus, ILTV, IBV, and infectious bursal disease virus (*Avibirnavirus gumboroense*) were identified as important. In the bovine sector, *S.* Dublin was mentioned by all participants. A common theme across several sectors was that providing data about genotyping for some pathogens is important (e.g., PRRSV). In addition, a need to build the dashboards in a format that makes it easy to add new pathogens was highlighted.

Disease (i.e., pathogen) frequency *forecasting* was identified as interesting, but as a future goal rather than the most immediate need.

### Description of test results for pathogens considered for the first iteration of dashboards

Overall, 42,961 unique submissions from livestock farms were submitted to the AHL between January 2013 and July 2022 to detect the poultry, swine, and bovine pathogens considered for dashboard development in our study. Of those, 26.1% were poultry submissions, 65.0% were swine submissions, and 9.0% were bovine submissions. In detail, 751 (6.7%) chicken submissions were tested for FAdV, 3,198 (28.6%) for IBV, 530 (4.7%) for ILTV, and 2,299 (20.5%) chicken and 4,418 (39.5%) turkey submissions were tested for IAV; 24,881 (89.2%) swine submissions were tested for PRRSV, 795 (2.8%) for rotavirus, and 2,232 (8.0%) for swine IAV; 2,757 (71.5%) bovine submissions were tested for BVDV, 461 (12.0%) for *M. bovis*, and 33 (0.9%; individual samples) and 606 (15.7%; individual samples) for *S.* Dublin. The reported results, converted into weekly time series, resulted in 499 observations that were used in designing dashboards.

**Figure 2.**
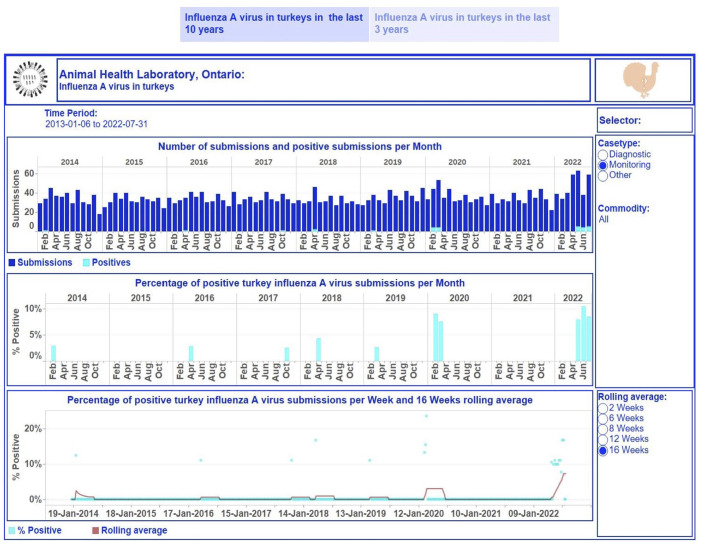
Dashboard for influenza A virus (IAV) in turkeys for January 2013 to July 2022 on a monthly basis. Top, stacked vertical bar graph: the number of unique monitoring submissions (dark-blue) and the number of unique positive monitoring submissions (light-blue) for IAV per month. Middle, vertical bar graph: the percentage of unique positive monitoring submissions for IAV per month. Bottom, dual graph: raw data of the percent positive time series per week (scatter plot in light-blue) and smoothed time series with a rolling 16-wk average (dark-red line). Middle, right side: selector of filtering options for users. Bottom, right: selection of time lags for rolling averages to smooth the percent-positive time series. Top right, turkey image: information regarding the tested turkey samples and explanations of performed calculations used to build graphics can be displayed by hovering over the image. Each visualization is embedded with a tooltip. By hovering over a mark, the tooltip displays relevant data and details from the visualizations specific to that mark.

**Figure 3.**
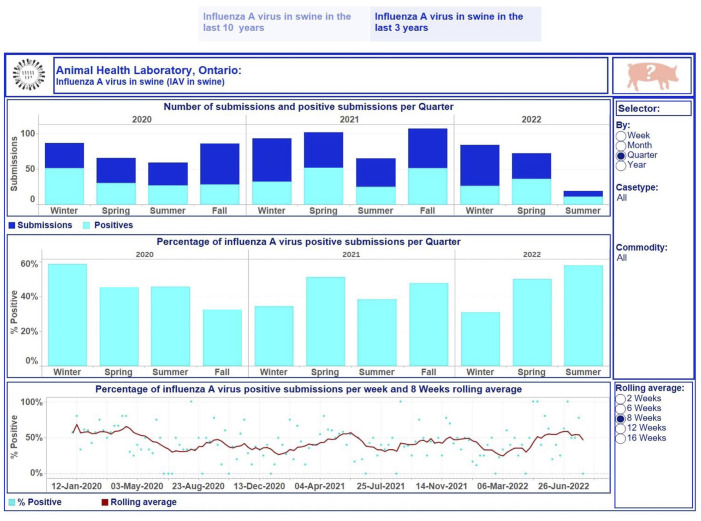
Dashboard for influenza A virus (IAV) in swine for January 2020 to July 2022 using quarter as the time aggregation unit. Top, stacked vertical bar graph: the number of unique submissions (dark-blue) and the number of unique positive submissions (light-blue) for IAV per quarter. Middle, vertical bar graph: the percentage of unique positive submissions for IAV per quarter. Bottom, dual graph: raw data of the percent positive time series per week (scatter plot in light-blue) and smoothed time series with a rolling 8-wk average (dark-red line). Middle, right side: selector of date level aggregation and filtering options for users. Bottom, right: selection of time lags for rolling averages to smooth the percent-positive time series. Top right, swine image: information regarding the tested swine samples and explanations of performed calculations used to build graphics can be displayed by hovering over the image. Each visualization is embedded with a tooltip. By hovering over a mark, the tooltip displays relevant data and details from the visualizations specific to that mark.

**Figure 4.**
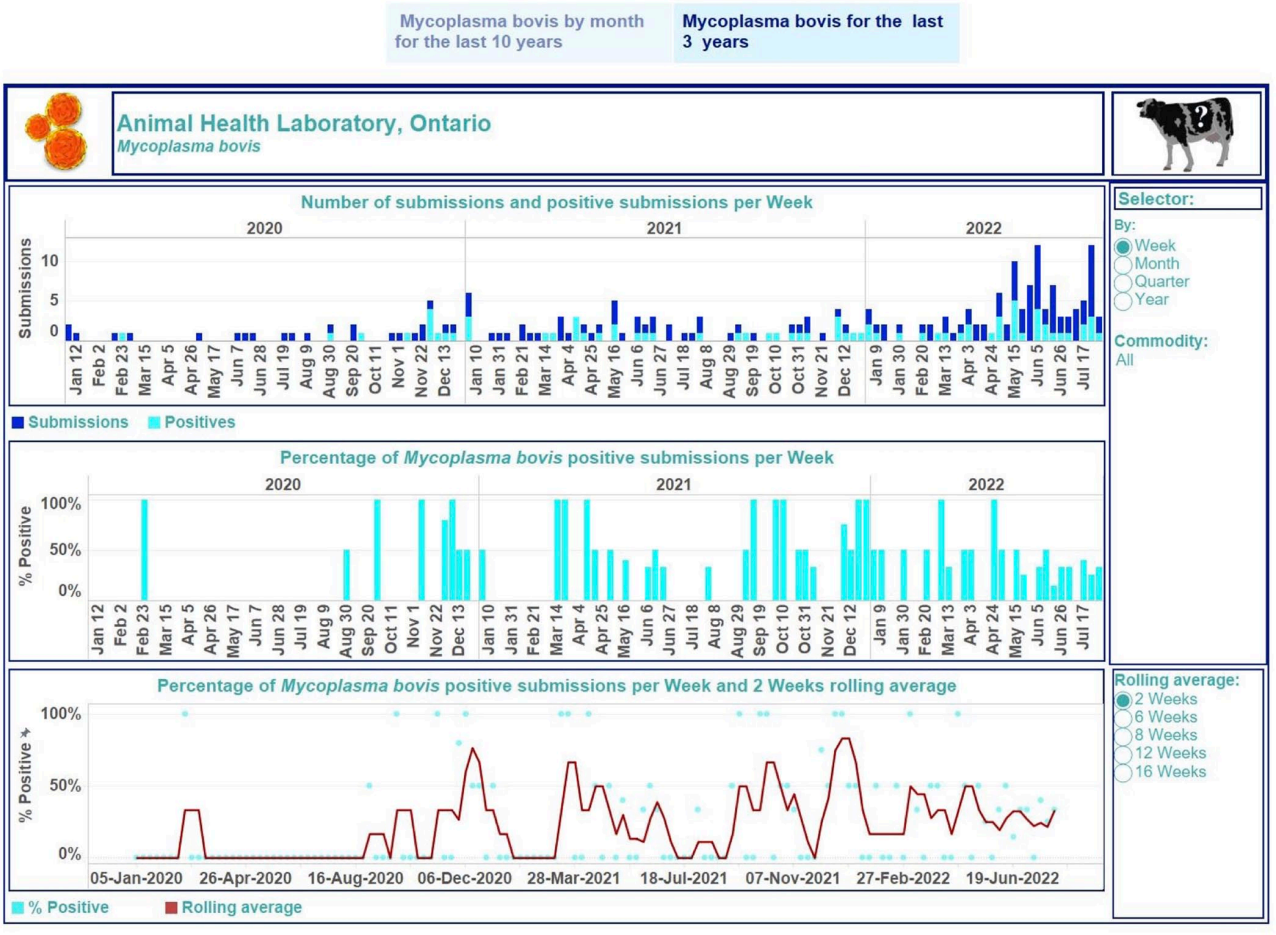
Dashboard for *Mycoplasma bovis* in cattle for January 2020 to July 2022 on a weekly basis. Top, stacked vertical bar graph: the number of unique submissions (dark-blue) and the number of unique positive submissions for *M. bovis* (light-blue) per month. Middle, vertical bar graph: the percentage of unique positive submissions for *M. bovis* per month. Bottom, dual graph: raw data of the percent-positive time series per week (scatter plot in light-blue) and smoothed time series with a rolling 2-wk average (dark-red line). Middle, right side: selector of filtering options for users. Bottom, right: selection of time lags for rolling averages to smooth the percent-positive time series. Top right, cow image: information regarding the tested animal samples and explanations of performed calculations used to build graphics can be displayed by hovering over the image. Each visualization is embedded with a tooltip. By hovering over a mark, the tooltip displays relevant data and details from the visualizations specific to that mark.

We presented the obtained outcomes in dashboards for different demographic or production commodities within a species (Figs. 2–4). For poultry, the commodity levels were exhibition or small farm flock and commercial farms. For swine, production commodities were suckling, nursery/weaner, grower/finisher, gilt/sow, boars, not specified, and not available or not provided (when the category of a host was not given). For cattle, the commodity levels were dairy, beef, and not specified or other (when a production category was not provided).

The first story point displays data insights across case type and commodity levels monthly from January 2013 to July 2022 (Fig. 2); the number of monitoring submissions from turkeys tested for IAV each month ranged from 18 to 63 submissions per month. The second story point presents results by case type and commodity levels at different time aggregations from January 2020 to July 2022 (Figs. 3, 4). The degree of smoothing short-term variation in the percent-positive time series is 16 wk. Results were summarized at quarterly intervals for submissions tested for IAV in swine (Fig. 3), which ranged from 19 to 107. The percent-positive time series was smoothed with a lag of 8 wk. Cattle submissions were tested for *M. bovis* weekly (range: 0–12 per wk; Fig. 4). A lag of 2 wk was selected to smooth the percent-positive time series. By visual inspection of the pathogen dashboards (Figs. 2–4), a user can view and compare results based on the selected filtering options.

The suggestion of the focus group participants to present a spatial distribution of cases by region was considered when dashboards were developed, taking into consideration data confidentiality, access to results by the end users, agreements to use existing data to generate maps, and data quality and quantity to allow such displays; spatial distribution was implemented for one dashboard (*S*. Dublin). The postal code of the submitting clinic was used to display a choropleth map of the numbers of submitted and positive submissions at the census division level.

## Discussion

Diagnostic laboratories are a valuable source of animal pathogen data. When such data are processed and displayed in a dashboard, the data can provide useful insights into disease trends for pathogens that are endemic (e.g., IAV in swine) or that re-emerge in susceptible populations (e.g., IAV in turkeys). Laboratory data from the AHL have been used to explore various questions including seasonality^21^ and forecasting capacity of different models^19^ for IAV in swine, the impact of COVID-19 on submission patterns for various pathogens,^20^ and the utility of laboratory submission data for syndromic surveillance,^8,9^ among others. The dashboards reported herein are a simple representation of temporal pathogen trends in Ontario and are targeted to provide near-real-time information to Ontario veterinary clinics that serve swine, bovine, or poultry production facilities, government, regulatory agency representatives, and researchers. Similar approaches have been reported in other jurisdictions for swine data and have been well-received by end users.^11^

The lag between the time that assay results are available in the AHL LIMS and the time when the aggregated results are displayed in dashboards is 24 h. Therefore, the end users of this information can have near-real-time descriptive data of trends for some of the most important pathogens, which could be further stratified by different temporal units, demographic data specific to the type of source population, and the reason for submitting samples. The reason for submission for most samples could be divided into 2 major categories: diagnostic and monitoring. Diagnostic submissions are more likely to be indicative of a clinical outbreak, whereas monitoring submissions, in general, are conducted for ongoing assessment of a circulating pathogen, or proof of negative status. Although the visualization of trends in diagnostic submissions could be suggestive of trends in clinical disease, their value should be interpreted with some caution for several reasons, including the following: 1) availability of the field for the reason for submission is directly linked with the type of submission form used when samples are submitted to the laboratory; 2) pathogens considered in these dashboards have different oversight from a veterinary regulatory perspective, which could influence the submission process itself and the selection of the choice of reason for submission by individuals or organizations submitting the samples; 3) interpretation of diagnostic versus monitoring submission may change over time as the needs of organizations for aggregated data interpretation change over time (this could influence sensitivity and specificity of the terms used); and 5) end-user preferences and habits when submitting samples. The degree to which the latter, and possibly other factors, play a role in specifying the reason for submission should be investigated further, ideally for each pathogen separately.

Focus group discussions allowed us to select and prioritize pathogens and types of analyses that were most useful and relevant to our target end users. Based on their feedback, we imported and analyzed data for the most recommended pathogens, resulting in 12 near-real-time dynamic and interactive dashboard stories, displaying primarily temporal trends.

For one pathogen (*S*. Dublin), the level of census division (CD) was selected as the geographic unit of interest for this project and is considered a compromise that would allow enough spatial granulation while maintaining the confidentiality of farm identity. The postal code of the clinic submitting the samples was considered a compromise solution in the absence of an accurate spatial identifier that could be easily incorporated into data-processing pipelines. The degree to which such a solution is a good approximation is unknown, and possibly varies depending on the current structure of farms in a specific sector and associated veterinary services.

The designs of the created dashboards were optimi-zed for desktop usage and were officially launched on 2022.01.12. End users can access the available visualizations on the Ontario IAPD website via a login account using their computers. Dashboard visualization layouts for other devices, such as phones and tablets, will be considered in the future.

The possible integration of weather and other publicly available data into the dashboards already developed will be considered in the future; the need for these features was not considered immediate. Furthermore, displaying genetic information is complex and specific for each pathogen and the type of genotyping approach employed; their detailed presentation is beyond the scope of our work and will be considered in the future.
